# The Lacanian Concept of Paranoia: An Historical Perspective

**DOI:** 10.3389/fpsyg.2017.01564

**Published:** 2017-09-15

**Authors:** Thomas Lepoutre, Manoel L. Madeira, Nicolas Guerin

**Affiliations:** ^1^Department of Clinical Psychology, Aix-Marseille University LPCLS (EA 3278) Marseille, France; ^2^Institute of Psychology, Federal University of Rio Grande do Sul Porto Alegre, Brazil

**Keywords:** DSM, foreclosure, Freud, Kraepelin, Lacan, nosography, paranoia, schizophrenia

## Abstract

This article seeks to reopen a major question raised by the Lacanian nosology of the psychoses, by looking closely at Lacan’s formulations of what he never ceased referring to as “paranoia”. While almost all classification systems of modern psychiatry, such as the ICD-10 and the DSM-5, have abandoned the specific category of paranoia, Lacan always viewed paranoia as a major category of “functional psychosis”. He held that paranoia was a qualitatively different disorder than schizophrenia, and considered it to be the principal or exemplary form of psychosis. Furthermore, in the middle period of his work, Lacan thought of paranoia in much broader terms than those of the definition proposed by Kraepelin, which he revisited, point by point, developing his theory of Freud’s concept of “*Verwerfung*” or foreclosure; the latter became the focal diagnostic criterion in his nosographic construction. Lacan’s privileging of and evolving theoretical views on paranoia provide a structural approach to what he called the “resistant nucleus” of psychosis; his work serves as a counterpoint to the more descriptive neo-Kraepelinian approach of contemporary psychiatric nosology.

## Introduction

This article seeks to reopen a major question raised by the Lacanian nosology of the psychoses, by looking closely at Lacan’s formulations of what he never ceased referring to as “paranoia”.

The need for such a reopening arises because successive editions of the principal international classification systems have ceased to use “paranoia” to delineate a unique diagnostic category, to the point that it seems destined to “fade away,” nosographically speaking (Prudent et al., 2016, p. 193). Lacan, on the contrary, used the concept intensively, so much so that he considered paranoia to be the prime example of psychosis. Has Lacan’s approach become outdated? Could it, on the contrary, remain of interest today, at least on the condition that certain of its subtleties are not overlooked? We shall argue for the second option.

To this end, we need to consider, jointly and separately, the *centrality* of the place accorded to paranoia in Lacan’s work and its increasing *marginalization* in contemporary nosography. We shall compare the slow incorporation of paranoia into the schizophrenia group (Marneros et al., 2012) with Lacan’s paradoxical consolidation of the concept of paranoia, a concept to which he frequently appealed as the exemplary form of psychosis throughout his rethinking of that problematic.

## Paranoia: Central for Psychoanalysis, Marginal for Psychiatry

Paranoia occupies an absolutely central place in Lacan’s work. From Lacan’s doctoral thesis on paranoiac psychosis written in 1932 (Lacan, 1975) and his first clinical publications in the 1930s (Lacan, 1931b, 1933/1988a, 1938/1988b), through his rereading of the Schreber case in 1955–1956 (Lacan, 1981/1993) and his programmatic text, “On a Question Prior to Any Possible Treatment of Psychosis,” of 1959 (Lacan, 1958/1966/2006b), to his much later work in the 1970s on knot theory, where it is reformulated and formalized in topological terms (Lacan, 2005/2016), paranoia was the clinical entity of reference for his approach to the clinical treatment of psychosis. Beyond its purely psychopathological denotations, psychosis is also the paradigmatic nosological form through which Lacan conceptualized a series of anthropological issues: the “hostile potentialities” that compose the social bond, for which the paranoid individual has an incomparable acumen (Lacan, 1975, p. 439); the problem of style in literary and artistic creation (Lacan, 1933); the “paranoiac structure of the ego” itself and the logic of analysis as a “guided paranoia” (Lacan, 1948/1966/2006a, pp. 93, 89); the recurring theme of “paranoiac knowledge” (cf. Lacan, 1947/1966/2006d, p. 150, Lacan, 1949/1966/2006e, p. 76); or again, in epistemological terms, what he called the “closure” of science (Lacan, 2017).

It is thus worthwhile to take a closer look at the reasons for Lacan’s apparent nosographical privileging of paranoia, both to identify its historical grounds and to grasp its clinical value. This is especially the case since, as a countercurrent to the Lacanian approach, the complex history of the category of paranoia – which has been at the heart of a certain number of terminological misunderstandings between European and American psychiatry (Pichot, 1997) – has led this term to be “overdiagnosed in the 19th century and underdiagnosed in the 20th” (Dowbiggin, 2000, p. 66). The shift in the frequency of diagnosis resulted from Kraepelin’s introduction of the category of “dementia praecox,” which Bleuler (1911/1950) soon renamed “schizophrenia”; this clinical entity came to absorb discussions of the “question of paranoia” or “*Paranoiafrage*” (Lewis, 1970, pp. 4–5) or to relegate them to the background. This led to Kraepelin’s redefinition of paranoia “in the strict or narrow sense [*im engsten Sinne*]” (Kraepelin, 1899a, p. 170). As a result, while Lacan’s writings attest to the centrality of paranoia for French psychoanalysts, modern psychiatrists, especially in North America, have tended to follow the opposite course, by underrating the nosological importance of paranoia and giving a hegemonic emphasis to schizophrenia (Kendler, 1982). The latter has been “broadened” and “expanded” (Andreasen, 1989, p. 520) to include paranoid forms, to the detriment of paranoia as a category.

Where does this sort of overrepresentation of paranoia in Lacan’s work come from? How did Lacan make the seemingly anachronistic choice to focus on paranoia, at a time when his contemporaries in psychiatry were concentrating on schizophrenia?

To reply to this question, we shall reassess how the theme of paranoia is situated with respect to Lacan’s theoretical path, which we view as being broadly organized into three major conceptual periods (Nobus, 2000; Vanheule, 2011). We shall concentrate on the first two of these, because they are decisive for the question of paranoia.

## Early Writings: Lacan With Kraepelin, 1931–1952

Although Lacan wrote several clinical articles about paranoia during the initial years of his work (Deloro, 2011), it is his doctoral thesis in medicine (Lacan, 1975), defended in 1932, that presents his first theoretical conception systematically.

In this thesis, Lacan referred to Bleuler, Kretschmer, and Jaspers to define paranoia as a “mode of reaction” (Lacan, 1975, p. 49), rather than as a developmental anomaly or an organic process; he intended to draw out the direct connection between the “events experienced” by the patient (p. 101), the ensuing “internal conflicts” (p. 277), and the psychotic break [*déclenchement*], understood as “the subject’s reaction to vital situations” (p. 77). His views were generally psychogenetic, and contrasted with the constitutionalist theories of Genil-Perrin and the organicist approach of Clérambault, which dominated psychiatry in France at the time. They also differed from Lacan’s own later views, when his “*return to Freud*” (Lacan, 1981/1993, p. 71) led him a position that was diametrically opposed to his initial “comprehensive” approach (p. 316).

Although writers such as Cox-Cameron (2000) and Vanheule (2011) have highlighted the implications of Lacan’s paradoxical and precocious early writings, it is noted less often that Lacan was also working to achieve a precise nosological definition and this led him to set out a lasting representation of the diagnostic concept of paranoia.

On this point, Kraepelin is the psychiatrist whom Lacan cites most frequently in his thesis, and whose work constitutes his major reference. After recalling the insoluble ambiguities and the “apex of the period of [nosological] confusion” around the *Paranoiafrage*, he concludes his history of the concept by noting, “*Then came Kraepelin, at last, as it were, with the Germanic clarity of his conceptions*”; for Lacan, Kraepelin’s definitions marked the “maturity of the work of delimitation as applied to the notion of paranoia” (Lacan, 1975, p. 23).

As Lacan notes (Lacan, 1975, p. 23), Kraepelin’s decisive move, which occurred in the sixth edition of his renowned *Lehrbuch*, consisted in “according a completely novel place to the concept of paranoia” (Kraepelin, 1899b, p. 430); in this, he diverged from traditional authors, who still associated paranoia with “a series of clinical pictures that no longer had any relation to the original concept, such as amentia, alcoholic hallucinations, and many other pathological states, which almost without doubt belong to dementia praecox or manic-depressive insanity” (p. 427).

This established the modern Kraepelinian concept (Kendler, 1988), to which those since Lacan have often returned, in which paranoia is limited to the “gradual development of a stable progressive system of delusions, without marked mental deterioration, clouding of consciousness, or disorder of thought, will, or conduct” (Kraepelin, 1899/1915, p. 423) – in opposition to the poor prognosis of the schizophrenia group.

Lacan refers to this “strict” Kraepelinian definition as “legitimate paranoia” (Lacan, 1975, p. 23). However, the definition, which is both the most orthodox psychiatric definition and one of the essential determining influences on how Lacan viewed paranoia, would be profoundly reworked in the course of his thorough rereading of Freud.

## The Return to Freud: Lacan With Schreber, 1953–1962

It is noteworthy that Lacan went on to revisit Kraepelin’s original definition of paranoia. Given his formulations in his thesis, it is even surprising that, 20 years later, Lacan opened his seminar on the psychoses (Lacan, 1981/1993) by going back to his critical point of departure and reworking the canonical definition, which has rarely been called into question by later psychiatrists, not even by members of the Saint Louis school (Winokur, 1977; Kendler, 1980, 1984) who instigated the neo-Kraepelinian revolution that inspired the DSM-III, DSM-IV, and DSM-5 revisions. Lacan actually repudiates Kraepelin’s definition in its entirety, to the point of stating that, “There isn’t a word of truth in it” (Lacan, 1981/1993, p. 17). He takes it apart point by point, casting doubt on each of its propositions successively: its supposedly gradual and continual development, the dependence of its etiology on purely endogenous causes, the illusory immutability of the system of delusions and the “total preservation of clarity and order in thought, will, and action” (p. 18).

Should we see in this apparent reversal a paradox in Lacan’s approach? While it could be thought that Lacan is quite simply going back to a pre-Kraepelinian conception, which he had rejected unambiguously at an earlier point, the situation turns out to be more complex.

On the one hand, several of Lacan’s criticisms had already been sketched out in his publications of the early 1930s.^1^ If he was able to express his criticisms more frankly in 1955, it might have been because his relation to psychiatry, and also to the rigors of pursuing a career within the hospital and university, was freer than it could have been in the 1930s. He could thus distance himself publicly from the authority figure of his thesis, and at a time when he had a robust conceptual framework that provided a new consistency to the different aspects of his critique of Kraepelin’s definition.

On the other hand, despite his thoroughgoing criticism, Lacan continued to praise Kraepelin’s conceptual prowess; this was still the case at the time of the publication of his *Écrits* in 1966, in which he reiterated his admiration for the German psychiatrist’s “clinical genius” and his “faithfulness to the symptom’s formal envelope” (Lacan, 1966/2006c, p. 52).

Nevertheless, alongside the methodical criticism intended to free the concept of paranoia from the “strict” psychiatric definition, Lacan’s response – by directing attention to questions of structure rather than to symptoms alone – had the effect of reopening the semiological framework of paranoia, to include “elementary phenomena” and the effects of “personal […] meaning” (Lacan, 1981/1993, pp. 19, 54) as well as verbal and auditory hallucinations. Lacan considered these to be the paradigms of paranoiac functions, whereas Kraepelin found they occurred “only quite occasionally” (Kraepelin, 1901/1904, p. 145).

The relaxing of the Kraepelinian frame was certainly consistent with Lacan’s overall conceptual development. Further, his emphasis on the structural analysis of “signifying effects” [*effets de signifiant*] in Freud’s case histories, and especially in the case of Schreber, ultimately led him, in this period of his work, to identify a particular mode of defense that would provide psychosis with its own specific etiology: the “foreclosure of the Name-of-the-Father,” which intervenes from the place of the Other (Lacan, 1958/1966/2006b, p. 477). The “failure of the paternal metaphor,” which serves as both the “essential condition” of psychosis and “the structure that separates it from neurosis,” has the necessary consequence of relegating the preoccupation with the nosography of paranoia to the background, for the sake of providing a clear characterization of psychotic structure as such (p. 479).

The downplaying of Lacan’s initial interest in differential categories, if not his discarding of a nosographical definition acquired with great effort during his thesis, derives, for the most part, from his deepening of the Freudian approach, to the detriment of psychiatry. This approach was reinforced by the contributions of linguistic theory, a theory that finally enabled him to give an epistemological heft to the term “structure,” which he had used more intuitively 20 years earlier.

Yet it is possible that Lacan was also taking his cues from another source. For a long time, Freud continued to privilege the earlier understanding of paranoia, rather than embracing the more modern dichotomy between paranoia and schizophrenia; this is shown by the many occasions in which he used these two terms as if they were interchangeable (Freud and Jung, 1974).

Freud did so because his deciphering of the clinical aspects of the psychoses was based on a pre-Kraepelinian nosography, with its broader and obsolete concept of paranoia. It can thus be noted that Freud’s first diagnosis of paranoia, which would inspire Lacan’s own concept of foreclosure, was explicitly corrected by Freud himself; once he had become aware of Kraepelin’s dismantling of the concept, he came to view the case as one of “dementia paranoides” (Freud, 1896/1962, p. 174, note 1, added in 1924; Freud, 1925/1959, p. 60). He was thus late to recognize Kraepelin’s revolution, for he only spoke of it in 1911: “I am of the opinion that Kraepelin was entirely justified in taking the step of separating off a large part of what had hitherto been called paranoia and merging it, together with catatonia and certain other forms of disease, into a new clinical unit…” (Freud, 1911/1958, p. 75). This rather laconic statement covers over a radical upheaval in the psychoanalytic nosography of the psychoses, through which it came to meet up with the modern psychiatric view that separates schizophrenia and paranoia, after long having allowed paranoia to occupy the nosological place of schizophrenia.

However, even after having imported Kraepelin’s categories into his own nosography as of 1911, Freud continued to use the concept of paranoia in its more extended sense, and this is what Lacan encountered. The ambiguity of Freud’s diagnosis of Schreber is a testament to this. Kraepelin, Bleuler, and Jung were unanimous in arguing that, as Jung put it, “Schreber’s case is not a pure paranoia in the modern sense” (Jung, 1912/1916, p. 510, note 8), but rather a case of dementia praecox (p. 141); Freud nevertheless retained his anachronistic diagnosis of paranoia. Lacan would then follow in his footsteps, as if taking no heed of the ambiguity of the double diagnosis that appears in the full title of Freud’s case history, which mentions both a “*Case of Paranoia*” and, parenthetically, “(*Dementia Paranoides*)”.

We can thus understand why Lacan began his seminar on the psychoses by emphasizing Freud’s privileging of paranoia:

In what has been done, is done, and is now in the course of being done concerning treatment of the psychoses the schizophrenias are much more readily explored than the paranoias, a much more lively interest is taken in them, and greater results are expected from this. Why then does paranoia, on the contrary, have a rather privileged position for Freudian doctrine – that of a knot, but also of a resistant nucleus? (Lacan, 1981/1993, p. 3).

What remains surprising is that, in noticing, supporting, and taking up this emphasis himself, Lacan fully recognizes the anachronism of that approach; he states expressly that he thinks that the historical reduction of the scope of paranoia had passed Freud by: “In this respect, as sometimes happens, Freud is not absolutely in step with his time. Is he way behind it? Is he way ahead of it? There lies the ambiguity. At first sight he is way behind it” (p. 4).

We see that Lacan was simultaneously emphasizing the central character of paranoia as a sort of historical scar and embracing it as a theoretical paradigm, as if he were hesitating between a purely historical privileging and a decidedly methodological preference…. In reality, Lacan was using what seemed to be a mere effect of history as a way of foreshadowing the structural framework that he was working to establish.

## Conclusion: Later Writings, 1962–1981

In his clinical approach to paranoia during the last period of his teaching, Lacan ultimately drew out its structural traits, thereby fully revealing, within the generic conceptualization of psychosis, the inadequacy of the distinctions between schizophrenia and paranoia that had been proposed before then.

If his return to Freud was primarily under the auspices of the logic of the signifier and lent support to the broad definition of paranoia, Lacan’s later work would focus instead on the limits of the symbolic, developing concepts related to jouissance and the object *a*. These concepts in turn had decisive effects on his ensuing work on psychosis in the 1960s, allowing him eventually to arrive at a “more precise definition of paranoia, as identifying jouissance in this place of the Other as such” (Lacan, 1966/2001, p. 215).

This fully fledged definition, with its structural criterion that is both more precise and extensive than our contemporary “diagnostic criteria,” also involves a complete parting of the ways between psychiatric and psychoanalytic nosographies. It does so by confirming the privilege of a definition that has been enlarged to cover all of what can be called the “elaborative psychoses,” which go far beyond the “delusional disorders” described in recent editions of the DSM. On this point, this new gulf between the structural definition provided by Lacanian psychoanalysis and the definition given by “neo-Kraepelinian” psychiatry seems, in the end, to renew and extend an earlier gulf: the one that had originally separated Freud’s initial broad definition from Kraepelin’s “strict sense” of the term.

For Freud, for whom paranoia, as he said in a letter to Jung, “remains the [major] theoretical concept” (Freud and Jung, 1974, p. 47), and for Lacan, for whom it constituted the most vivid clinical form of the foreclosure of the paternal function, paranoia continued to be the “resistant nucleus”. Perhaps psychoanalysis may be thought, in some sense, to play a similar role for clinical psychiatry.

## Author Contributions

All authors of this article certify that they each have provided substantial contributions to the conception or design of this work or the acquisition, analysis, or interpretation of data for the work; have participated in drafting the work or revising it critically for important intellectual content; have provided final approval of the version to be published; and agree to be accountable for all aspects of the work in ensuring that questions related to the accuracy or integrity of any part of the work are appropriately investigated and resolved.

## Conflict of Interest Statement

The authors declare that the research was conducted in the absence of any commercial or financial relationships that could be construed as a potential conflict of interest.

## Footnotes

1 The discontinuous or episodic progress of the disorder was emphasized in Lacan’s article on “schizography” (Lacan, 1931a); the basing of the etiology on external or social and intersubjective, rather than internal, causes, was developed in several works of the period (Lacan, 1931b, 1933, 1933/1988a) as well as in the 1932 thesis (Lacan, 1975). Lacan had also already begun to question the supposed “total preservation of clarity and order in thought” in his thesis, in relation to the case of Aimée and the retroactive reconstruction of the past imposed by the dynamic of the delusion on the subject’s relation to her history.
